# Prediction equations of forced oscillation technique: the insidious role of collinearity

**DOI:** 10.1186/s12931-018-0745-8

**Published:** 2018-03-27

**Authors:** Hassib Narchi, Afaf AlBlooshi

**Affiliations:** Department of Pediatrics, College of Medicine and Health Sciences, United Arab Emirates University, Al Ain, P O Box 17666, United Arab Emirates

**Keywords:** Collinearity, Multicollinearity, Multivariable models, Forced oscillation technique

## Abstract

Many studies have reported reference data for forced oscillation technique (FOT) in healthy children. The prediction equation of FOT parameters were derived from a multivariable regression model examining the effect of age, gender, weight and height on each parameter. As many of these variables are likely to be correlated, collinearity might have affected the accuracy of the model, potentially resulting in misleading, erroneous or difficult to interpret conclusions.

The aim of this work was: To review all FOT publications in children since 2005 to analyze whether collinearity was considered in the construction of the published prediction equations. Then to compare these prediction equations with our own study. And to analyse, in our study, how collinearity between the explanatory variables might affect the predicted equations if it was not considered in the model. The results showed that none of the ten reviewed studies had stated whether collinearity was checked for. Half of the reports had also included in their equations variables which are physiologically correlated, such as age, weight and height. The predicted resistance varied by up to 28% amongst these studies. And in our study, multicollinearity was identified between the explanatory variables initially considered for the regression model (age, weight and height). Ignoring it would have resulted in inaccuracies in the coefficients of the equation, their signs (positive or negative), their 95% confidence intervals, their significance level and the model goodness of fit. In Conclusion with inaccurately constructed and improperly reported models, understanding the results and reproducing the models for future research might be compromised.

## Introduction

While spirometry is the gold standard method for assessing lung function in older children and adults, performing it is not possible when a forced expiratory maneuver cannot be successfully achieved, such as in young children [1]. The forced oscillation technique (FOT), used in children, measures, at different frequencies, the mechanical behaviour of the respiratory system, including resistance (Rrs), reactance (Xrs), resonance frequency (Fres), frequency dependence (Fdep) and the area under reactance curve (AX) [2–4]. As all these measures change physiologically with growth, reporting their Z score values facilitates their interpretation across a wide range of anthropometric measures throughout childhood [5–7]. Establishing reference data for FOT in children is, therefore, essential.

Many studies have already reported FOT reference data in healthy children as a function of several factors, such as age, weight, height, gender, ethnicity and equipment used [8–17]. In all these studies, the prediction equation of each FOT parameter was derived from a multivariable regression model that included one or more of the following explanatory variables: age, gender, weight and height. The significant coefficients of these models were used to build the respective prediction equations [5, 6].

All multivariable modeling strategies have strict assumptions and several limitations which, when violated, may affect the accuracy of the results, their proper interpretation by the reader and their use in clinical care and in future research [18–25]. The majority of clinicians and researchers rely on the editorial and peer review processes of the journals to ensure that the statistical methods in the articles have been appropriately used and correctly interpreted [26–28].

One assumption, often neglected in multivariable regression models, is collinearity. It occurs whenever there is a high correlation between two explanatory variables and is called multicollinearity in case of correlations between three or more variables. Both terms will be used interchangeably in the text. Collinearity creates very unstable estimated regression coefficients caused by redundant information, because the effect of correlated variables overlaps, making it impossible to accurately estimate the independent effect that each variable has on the studied outcome. It affects the estimations of individual predictors because the coefficient estimates will change erratically in response to small changes in the model or the data [29]. Collinearity also inflates the standard errors of these estimates, causing inaccurate and inflated variances. This affects the reliability of the confidence intervals estimation and leads to incorrect inferences about relationships between explanatory and response variables. Variables with no significant relationship with the outcome, when considered in isolation, might then become highly significant when considered in conjunction with collinear variables, resulting in an increased risk of false-positive results (Type I error). Similarly, several coefficients might show no statistical significance due to incorrectly estimated wide confidence intervals, resulting in an increased risk of false-negative results (Type II error). Furthermore, although the collinear variables may sometimes remain statistically significant, the sign of their regression coefficient might be the reverse of what would be expected (from positive to negative coefficients, or vice-versa) [30]. Thus, erroneous conclusions might be drawn about the relationships between explanatory and response variables. Although the reporting quality and reliability of models constructed by researchers can always be improved by editorial and peer review processes, as well as a statistical review system, [26, 31] collinearity is often ignored as studies have shown that it is systematically checked in only 1–2% of published articles [27, 28].

In this study, we aimed to evaluate the role of collinearity in previously published pediatric FOT reference articles which are often cited in most manuscripts. We reviewed several publications in children since 2005, to estimate if collinearity had been taken into consideration before modeling and reporting the predictive regression equations. Furthermore, to illustrate the impact of collinearity on the interpretation of the coefficients in such models, we also analyzed, hypothetically, the effect that collinearity might have had on the findings in our own report in which we constructed FOT reference data for children in our community (*AlBlooshi, unpublished data*).

## Methods

1. We reviewed the published literature of the predictive equations of FOT airway resistance (Rrs) since 2005. These equations were constructed from the coefficients of a multivariable model that had included age, weight and height as explanatory variables. We report which of these variables were included in the model, if collinearity was explicitly checked for or not, and if present, what measures were taken to improve the reported coefficients. As the frequencies at which airway resistance was measured varied with the FOT commercial equipment used in each report, but were quite similar between Rrs5, Rrs6 and Rrs8, we compared the resistance measured in this narrow frequency range.
2. Using the age range of participants in each report, we compared airway resistances that would have been predicted, based on height, if these equations were applied to a cohort of 291 children (aged between four and 12 years) in our own study, approved by our institution’s ethics committee (Ref. DT/bb/15–32) (*AlBlooshi, unpublished).* As the units in which resistance was expressed were not similar amongst those reports, we reported them all in *cm H*_*2*_*O.s.L*^*− 1*^ to allow comparison.
3. We studied the effect of collinearity on the coefficients of the variables used in our own study (described above) (*AlBlooshi, unpublished*). We analyzed first the Pearson correlation coefficients (r) between the explanatory variables (age, weight and height) used to predict the resistance value Rrs5. We modeled separately several regression equations which included one or more of these variables. We separately calculated, for each model, the constant value, the respective coefficient of each variable in the model, with its 95% confidence intervals, the *P* value as well as the standard error of the equation (SEE) and the goodness of fit of the model (R^2^). We also calculated, for each model, the respective centered variance inflation factor (VIF) for each explanatory variable. We then compared the coefficients of the explanatory variables in all the models. We also displayed graphically the predicted Rrs5 between these hypothetical models, to visualize the effect of multicollinearity on the predicted plots, looking for any overlapping, convergence or divergence of these plots.

All analyses were performed with the statistical package STATA version 14 (StataCorp, College Station, TX, USA).

## Results

### Previous studies

We reviewed ten published studies on FOT reference data in children since 2005 [8–17]. (Table 1). None of the studies had explicitly stated in the methods if collinearity was checked for. Furthermore, five studies (50%) had included, in their equations, variables that are biologically and physiologically correlated, such as age, weight and height [8, 10, 15–17].

**Table 1:** Comparison of published equations since 2005 of airway resistance (Rrs) at 5 or 6 or 8 Hz with the explanatory variables used in their respective regression model

Authors (reference)	Ethnic group	Equipment used	Subject number	Age range Years	Reported resistance	Variables entered in the model	Indication if collinearity was considered
Frei [12]	Canadian	IOS Jaeger	222	3–10	Rrs5	Height	No
Hall [14]	Australian	FOT, I2M	158	2–7	Rrs6	Height	No
Vu [13]	Vietnamese	FOT, Pulmosfor	175	6–11	Rrs8	Height	No
Calogero [9]	Italian	FOT, I2M	163	2–6	Rrs6	Height	No
Shackleton [11]	Mexican	FOT, I2M	584	3–5	Rrs6	Height	No
Dencker [10]	Scandinavian	IOS Jaeger	360	2–11	Rrs5	Height	No
						Weight	
Nowowiejska [16]	Polish	IOS Jaeger	626	3–18	Rrs5	Height	No
Park [17]	Korean	IOS Jaeger	119	3–6	Rrs5	Height	No
						Gender	
Calogero [8]	Italian and Australian	FOT, I2M	760	2–13	Rrs6	Height	No
						Gender	
Amra [15]	Iranian	IOS Jaeger	509	5–18	Rrs5	Height	No
						Weight	
						Age	

*Rrs* resistance, *IOS* impulse oscillation system, *FOT* forced oscillation technique

### Comparison of predicted resistance amongst the studies

**Fig. 1:**
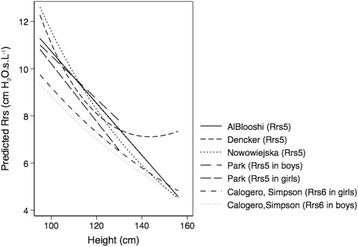
Comparison of the predicted airway resistance (Rrs) by height between our study with an equation based exclusively on height (*ABlooshi, unpublished*) and reports that included other correlated variables in their respective equations (Dencker [10], Nowowiejska [16], Park [17], Calogero [8])

The predicted airway resistance values (Rrs5, Rrs6) were compared between our study with an equation based exclusively on height (*AlBlooshi, unpublished)* and the reports that had included other correlated variables to their respective equation (Fig. 1). Across the children’s height range, there were differences in the predicted resistance amongst all the equations. For example, for a child of a height of 120 cm, while our equation predicts Rrs5 of 9.50 cm H_2_O.s.L^− 1^, the predicted values from the other equations ranged from 7.00 to 9.00 cm H_2_O.s.L^− 1^, a difference of 28.6%. Furthermore, except for one equation (Dencker [10]), the variation in the predicted resistance amongst the different equations decreased with increasing height.

### Our study

**Fig. 2:**
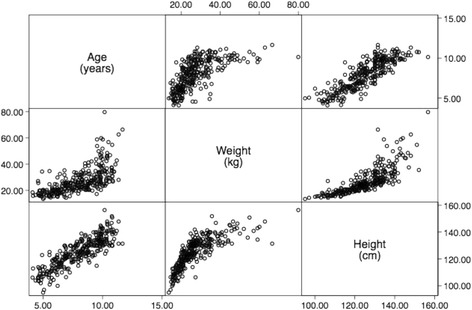
Scatterplot matrix of the explanatory variables initially considered in the multivariable linear regression model of resistance (Rrs5) in 291 healthy children (AlBlooshi, et al. *unpublished data*). r = Pearson correlation coefficients (r) between the 3 explanatory variables

In our own study *(AlBlooshi, unpublished*) we enrolled 291 children with an age ranging from four to 12 years and not known to have any respiratory problem. The Pearson correlation coefficients (r) between the explanatory variables that we had initially considered (age, weight and height) showed a positive and strong correlation (0.65 to 0.85), indicating a significant collinearity between them, defining multicollinearity (Fig. 2).

**Fig. 3:**
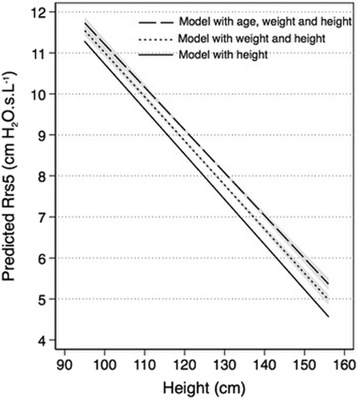
Comparison of the predicted linear equations slopes of resistance (Rrs5) expressed as cm H_2_O.s.L^− 1^, with their 95% confidence intervals (shaded zones), amongst three multivariable linear regression models with inclusion of different collinear explanatory variables, in 291 healthy children (AlBlooshi, et al. *unpublished data*)

The coefficients of the explanatory variables, their signs (positive or negative), their 95% confidence intervals, their significance level and the model goodness of fit varied significantly when the model included collinear variables and when this was avoided (Table 2). The changes in the direction (positive versus negative) of the coefficients of the same variables across the different tested models was also highly suggestive of collinearity. In the model including the collinear variables, the centered VIF values of most coefficients was > 2.5 and their average (3.3) was significantly higher than one, also suggesting the presence of collinearity between the respective variables. A graphical comparison of the predicted resistance Rrs5 between three hypothetical linear regression models which included different explanatory variables showed significant divergence in the curves (Fig. 3). The more collinear variables were included in the model, the higher the Rrs5 values were predicted for the same height. The respective 95% confidence intervals of the three linear curves showed no overlap. This divergence in the predicted Rrs5 values by the three models increased gradually with increasing height. Taking the same hypothetical example of a child with a height of 120 cm, the predicted Rrs5 value would have varied from 8.5 cm H_2_O.s.L^− 1^ in the equation including only height, to 9.5 cm H_2_O.s.L^− 1^ in a model using three collinear variables, a difference of 11.7% (Fig. 3).

**Table 2:** Comparison of the effect of collinearity on the coefficients of the equation developed on 291 healthy children for airway resistance (Rrs5) using a multivariable linear regression model (*AlBlooshi, unpublished*), with resistance expressed as cm H_2_O.s.L^−1^

Constant	Variables (units)	Coefficient	95% CI^a^	*P* value	VIF^b^	SEE^c^	R^2^	Collinearity adjusted for
23.51	Age	−0.19	−0.37 to − 0.006	0.04	3.6	1.54	0.41	No
	Height	−0.12	− 0.16 to − 0.08	< 0.001	5.4			
	Weight	−0.06	0.03 to 0.09	< 0.001	2.6			
12.78	Age	−0.64	−0.77 to − 0.50	< 0.001	1.74	1.65	0.32	Height excluded
	Weight	0.002	−0.02 to 0.02	0.8	1.74			
19.71	Age	−0.20	−0.39 to − 0.02	0.03	3.6	1.58	0.38	Weight excluded
	Height	−0.08	− 0.11 to − 0.05	< 0.001	3.6			
25.45	Height	−0.15	− 018 to − 0.12	< 0.001	2.6	1.55	0.40	Age excluded
	Weight	0.06	0.03 to 0.09	< 0.001	2.6			
12.77	Age	−0.62	−0.73 to − 0.52	< 0.001	1.0	1.65	0.32	Yes
9.76	Weight	−0.07	− 0.09 to − 0.05	< 0.001	1.0	1.87	0.13	Yes
21.73	Height	−0.11	−0.12 to − 0.09	< 0.001	1.0	1.59	0.37	Yes^d^

^a^Confidence interval, ^b^ Variance inflation factor, ^c^ Standard error of the equation; ^d^ Model finally retained

## Discussion

The observed differences in the results between these studies which we have compared can be attributed to the use of different equipment for FOT measurements, using differing characteristics of perturbation signals of impulse oscillometry versus composite sinusoidal FOT signaling, for example (Table 1). This, however, was not the main purpose of our study. Our aim was to demonstrate that, if collinearity is not considered in a study, the resulting prediction equation obtained using any equipment may be incorrect, as illustrated with our own data. This error may inflate further the differences observed between studies using different equipment.

None of the ten reviewed studies had stated if collinearity was checked for, confirming prior reports [27, 28]. We were, however, unable to determine whether the statistical analyses were incorrect, if the authors had deemed unnecessary to check for collinearity, if they had valid but undeclared reasons to make exemptions or simply reported an incomplete methodology. Of concern, however, is that half of those reviewed reports still included in their equations explanatory variables which are physiologically correlated, such as age, weight and height. Clearly, it is biologically implausible if these three variables were not correlated. Depending on which published equation a clinician may use, a 28% difference in the predicted Rrs may occur with potential impact on the quality of care.

In our own study, we found multicollinearity between the explanatory variables initially considered for the regression model (age, weight and height). Its effects included the wide variations in the coefficients of the explanatory variables, their changing signs (positive or negative), their wide confidence intervals, their changing significance level and the different results of the model goodness of fit obtained by the different hypothetical models. In addition, the centered VIF values of most coefficients was > 2.5, with an average of 3.3, significantly higher than one, constituting further evidence of collinearity in the models [32]. A 11.7% difference in the predicted Rrs in our population, depending on the model in use, with and without collinear variables, cannot be inconsequential.

As there is no automatic warning of the presence of multicollinearity in many statistical packages, it is necessary for the researchers to check for it systematically before constructing multivariable models. Several methods exist to identify multicollinearity. A simple rule of thumb is to first test the explanatory variables for correlation. Another commonly used measure is the variance inflation factor (VIF), defined as VIF = 1/(1-R^2i^) where R^2i^ is the R^2^ for a covariate x^i^ regressed on the remaining covariates in a separate regression. It indicates the strength of the dependencies and quantifies the collinearity-induced inflation of the variances of each regression coefficient compared to when the independent variables are not correlated. Although there are no formal rules, it is generally accepted that a VIF value exceeding 10 is often regarded as indicating multicollinearity, while values above 2.5 should also be a cause for concern [33, 34]. Unexpected changes in the direction of association between the outcome and an explanatory variable (from positive to negative coefficient, or vice-versa) is also a common result of collinearity [35].

To avoid the detrimental effects of collinearity on a regression mode, several methods have been suggested. Redundant collinear or duplicate explanatory variables are often removed [36, 37]. Collinear variables can also be combined into a single index. One method is centering, which involves the creation of a new covariate or an interaction term (usually by multiplication) between two collinear variables, after having centered their initial values (i.e. transforming them by subtracting the calculated mean from their individual value) [29, 35]. Principal component analysis (PCA), or factor analysis, is also useful to eliminate the effect of multi-collinearity and also to eliminate the indirect effect of imperfect parameters [29, 38].

## Conclusion and recommendations

An improvement in the construction and reporting of multivariable regression models would undoubtedly help the reader in appropriately interpreting the data. Researchers should systematically adopt robust diagnostics for collinearity, report them and use appropriate procedures to eliminate them, prior to constructing the final model and establishing the predictive equation coefficients. A closer cooperation with statisticians and epidemiologists would be very constructive in that regard. Journals should also develop statistical reporting guidelines concerning multivariate regression models [26, 31, 39]. The regression models and their results in the submitted manuscripts should be verified at the editorial level, by the peer reviewers and also require a formal statistical review [26, 31]. The accurate, reliable and responsible transmission of scientific knowledge from the researcher to the reader requires no less.

## Abbreviations

AX: Area under reactance curve
CI: Confidence interval
Fdep: Frequency dependence
FOT: Forced oscillation technique
Fres: Resonance frequency
PCA: Principal component analysis
r: Pearson correlation coefficient
R2: Goodness of fit of the model
Rrs: Resistance
Rrs5: Resistance at 5 hertz
Rrs6: Resistance at 6 hertz
Rrs8: Resistance at 8 hertz
SEE: Standard error of the equation
VIF: Variance inflation factor
Xrs: Reactance
